# Global Emergence and Spread of the XFG SARS-CoV-2 Variant and Its Clinical Profile Among XFG Patients in Maharashtra, India

**DOI:** 10.7759/cureus.103574

**Published:** 2026-02-14

**Authors:** Rajesh P Karyakarte, Rashmita Das, Sushma Yanamandra, Kalyani Jagarwal, Preeti Pawar, Viswanathan D V, Gilbert Chamy, Vasundhara Singh, Anna Tete, Asawari Koshti, Damini More, Poonam Madne, Vandana Patil, Suvarna Joshi, Jyoti Gurav

**Affiliations:** 1 Microbiology, Byramjee Jeejeebhoy Government Medical College and Sassoon General Hospitals, Pune, IND; 2 Integrated Disease Surveillance Programme, Directorate of Health Services, Government of Maharashtra, Pune, IND

**Keywords:** clinical characteristics, covid-19, sars-cov-2, variant under monitoring (vum), xfg

## Abstract

Background: In early 2025, changing dominance among Omicron-derived lineages culminated in the emergence of the recombinant XFG variant. XFG exhibited a significant growth advantage, rapidly emerging as the predominant SARS-CoV-2 variant in India and globally, prompting its designation as a Variant Under Monitoring by the World Health Organization in June 2025. This study examines the spread of the XFG variant and describes its clinical characteristics in Maharashtra, India.

Methodology: The dataset included 204,603 SARS-CoV-2 genomes from the Global Initiative on Sharing All Influenza Data (GISAID) and 1,089 XFG sequences from India (obtained from the Indian Biological Data Centre (IBDC)), collected between November 1, 2024, and October 31, 2025, to assess the temporal and geographic distribution of XFG in the context of global SARS-CoV-2 circulation. Telephonic interviews were conducted to obtain clinical information for the XFG cases in Maharashtra. All data were recorded and analyzed using Microsoft® Excel (Microsoft Corporation, Redmond, Washington, United States).

Results: Among 204,603 global SARS-CoV-2 genomes analyzed, XEC (20.4%) and XFG (18.8%) were the most prevalent lineages, with XFG emerging as a major global driver during mid-to-late 2025. XFG became dominant in North America, Europe, South America, and Africa, accounting for 66-83% of late-phase sequences, while remaining less prevalent in Asia and Oceania (<20%). Globally, the distribution of XFG sublineages was dominated by the parent lineage XFG (71.1%), followed by XFG.3 (12.6%), XFG.1 (5.8%), XFG.3.4.3 (5.4%), XFG.3.4 (3.3%), and XFG.2 (1.8%). In India, among the 1,089 XFG sequences, 27 sublineages were identified, predominantly XFG.3 (45.27%) and XFG.4 (41.78%), with the highest contributions from Gujarat, Maharashtra, West Bengal, and Rajasthan. Maharashtra reported its first XFG detection in early January 2025, followed by a sharp surge during May-June 2025. Clinical evaluation of 99 laboratory-confirmed XFG cases from Maharashtra showed that most patients were symptomatic (92.93%) but experienced predominantly mild illness, commonly presenting with fever, cough, cold, body ache, fatigue, and sore throat. Comorbidities were present in 31.31% of cases, most frequently diabetes mellitus and hypertension. Most patients were managed at home (68.69%), while 31.31% required hospitalization; among these, 61.29% received conservative care, and 38.71% required predominantly non-invasive oxygen support. Vaccination coverage was high (90.91%), with all unvaccinated cases occurring in children aged 0-9 years. Overall outcomes were favorable, with a recovery rate of 95.96% and low mortality (4.04%), confined to elderly patients with significant comorbidities.

Conclusion: The XFG variant emerged as the dominant SARS-CoV-2 lineage globally during late 2024-2025 and circulated widely across India, with a brief mid-2025 surge. Despite reduced clinical severity, XFG's rapid spread and diversification highlight ongoing viral adaptability and the need for strengthened, complementary surveillance to detect emerging variants early.

## Introduction

SARS-CoV-2, the virus responsible for COVID-19, continues to circulate globally and evolve since its first emergence in late 2019. Its remarkable adaptability is evident from the steady accumulation of a large number of mutations and emergence of multiple lineages and sublineages with varying transmission capabilities [1]. During the period from January to May 2025, the global SARS-CoV-2 variant dynamics underwent notable shifts. At the beginning of the year, XEC was the most prevalent variant globally, followed by KP.3.1.1. By February, XEC circulation began to decline, whereas LP.8.1 started to rise and became the most commonly detected variant by mid-March 2025. From mid-April, LP.8.1 started to decline with NB.1.8.1 detection increasing steadily [2]. 

Early 2025 saw a notable uptick in COVID-19 activity across parts of the Eastern Mediterranean, Southeast Asia, and Western Pacific regions [2]. Within Southeast Asia, a marked increase in cases was reported particularly in China, Hong Kong, Singapore, Thailand, and India [3]. This surge, beginning in late April and early May 2025, was primarily driven by the NB.1.8.1, a descendant of XDV.1.5.1, which evolved from the JN.1 variant [3,2]. As NB.1.8.1 continued to rise in global prevalence, it was designated as a Variant Under Monitoring (VUM) on May 23, 2025, by the World Health Organization (WHO), with the earliest sample collected on January 22, 2025 [4].

During this period of increasing transmission, a newly identified recombinant lineage, XFG, began to gain prominence and rapidly overtook NB.1.8.1 in circulation. This variant emerged through recombination between the Omicron sublineages LF.7 and LP.8.1.2, with the earliest sample collected on January 27, 2025. By late June 2025 (epidemiological week 22), the XFG variant had been detected in 38 countries and accounted for 22.7% of all global sequences submitted to the Global Initiative on Sharing All Influenza Data (GISAID). Its expansion was particularly striking in the Southeast Asia region, where its prevalence rose from 17.3% to 68.7%, following an earlier period of rapid dominance by NB.1.8.1 in the spring. On June 25, 2025, the WHO designated XFG as a VUM due to its apparent growth advantage over other circulating variants, including NB.1.8.1. Notably, in India, XFG had already established itself as the predominant lineage during the spring months, while NB.1.8.1 remained uncommon [5].

The present study aims to describe the temporal trends of the XFG SARS-CoV-2 variant globally and within India and to compare the trends in the context of the variant's broader emergence. In addition, it characterizes the clinical characteristics of laboratory-confirmed XFG cases in Maharashtra, India, and analyzes the associations between clinical variables and disease severity. Together, these analyses offer a comprehensive understanding of the public health relevance and clinical impact of this emerging variant.

## Materials and methods

The present study forms a part of ongoing SARS-CoV-2 genomic surveillance efforts in Maharashtra, under the framework of the Indian SARS-CoV-2 Genomics Consortium (INSACOG), with the objective of monitoring genetic diversity and evolutionary trends of SARS-CoV-2.

This study was an observational, retrospective study analyzing the SARS-CoV-2 surveillance data. It comprised a descriptive analysis of publicly available SARS-CoV-2 sequences from GISAID and the Indian Biological Data Centre (IBDC) to describe temporal and geographic trends of XFG and a cross-sectional clinical characterization of laboratory-confirmed XFG cases in Maharashtra using telephonic interviews. The study was conducted at the Department of Microbiology, Byramjee Jeejeebhoy Government Medical College and Sassoon General Hospitals, Pune, India, after obtaining approval from the institute's Institutional Ethics Committee (approval number: BJGMC/IEC/Pharmac/ND-Dept0721233-233).

Lineage analysis of SARS-CoV-2 whole genome sequences from India and globally

To characterize the emergence and distribution of the XFG SARS-CoV-2 variant in Maharashtra, India, and beyond, publicly available SARS-CoV-2 genome sequences collected between November 1, 2024, and October 31, 2025, were analyzed. Genome data generated and submitted by various regional and national sequencing laboratories across India were accessed, with necessary permissions, from the Indian Biological Data Centre (IBDC). In parallel, genome sequences submitted by various international laboratories were retrieved from the GISAID repository [6]. Only complete sequences (sequences exceeding 29,000 nucleotides in length) accompanied by complete metadata, including sampling date and geographical origin, were included in the final dataset. Entries with low coverage (>5% Ns) were excluded from the dataset. The details of sequences retrieved from GISAID and IBDC are provided in Appendix A and Appendix B, respectively.

Lineage and clade annotations provided by GISAID and IBDC were utilized to describe the temporal and geographic distribution of the XFG sequences from India and of all circulating SARS-CoV-2 variants from the global dataset.

Collection of demographic and clinical data of XFG SARS-CoV-2-positive cases in Maharashtra

Demographic information, including age, sex, area of residence, contact details, and dates of sample collection, was compiled for XFG-positive cases sequenced at the Department of Microbiology, Byramjee Jeejeebhoy Government Medical College and Sassoon General Hospitals, Pune, Maharashtra, India. These details were extracted from the metadata provided by reverse transcription-polymerase chain reaction (RT-PCR) testing centers along with the specimens submitted for sequencing. To verify these records, telephonic interviews were conducted with individual patients after obtaining verbal consent. During these interviews, additional clinical information, such as presenting symptoms, type of isolation, hospitalization history, oxygen requirement, treatment received, and vaccination status, was collected. Responses were primarily self-reported; however, for children or severely ill individuals unable to respond, information provided by the primary caregiver was accepted and recorded. Cases where patients declined to share clinical details were documented and subsequently excluded from the analysis. The questionnaire template used in the study is available in Appendix C.

Daily epidemiological data, including the number of SARS-CoV-2 tests performed, test positivity rates, and counts of recoveries and deaths across Maharashtra, were obtained from the State District Health Services Department for the period between November 2024 and September 10, 2025. These data were analyzed to evaluate potential case surges temporally associated with the emergence of the XFG SARS-CoV-2 variant in the state.

Statistical analysis 

Demographic and clinical datasets were recorded and analyzed using Microsoft® Excel (Microsoft Corporation, Redmond, Washington, United States). Continuous variables were presented as medians with corresponding interquartile ranges (IQRs), while categorical variables were reported as frequencies and percentages.

To assess the temporal changes in SARS-CoV-2 positivity, positivity rates were compared across aggregated epidemiological periods (pre-peak: November 2024 to April 2025; peak: May to July 2025; post-peak: August to September 2025) using the chi-squared test. Bivariate and multivariate analyses were performed to evaluate associations between clinical variables and disease severity, using hospitalization and mortality as outcomes. A p-value of ≤0.05 was considered statistically significant.

## Results

Overview of global SARS-CoV-2 variant landscape

A total of 204,603 SARS-CoV-2 whole-genome sequences, submitted to GISAID during the study period, were analyzed to characterize the global distribution of major circulating lineages. The data reflects substantial lineage diversity worldwide, with over a thousand (1001) Omicron-descendant variants, including prominent variants such as XEC, XFG, MC, KP, LP, NB.1.8, XDV, JN, and LF and several other recombinant lineages.

Globally, XEC was the most dominant lineage detected (41,824 sequences; 20.4%), closely followed by XFG (38,435 sequences; 18.8%) and MC (20,450 sequences; 10%). Other notable contributors included KP (14,526 sequences; 7.1%), LP (13,863 sequences; 6.8%), JN (12,698 sequences; 6.2%), NB.1.8 (11,035 sequences; 5.4%), and XDV (10,984 sequences; 5.4%). A broad array of minor and other variants made up the remaining 46.7% (95,650 sequences), while other recombinant variants comprised 3.3% (6,674 sequences).

**Figure 1:**
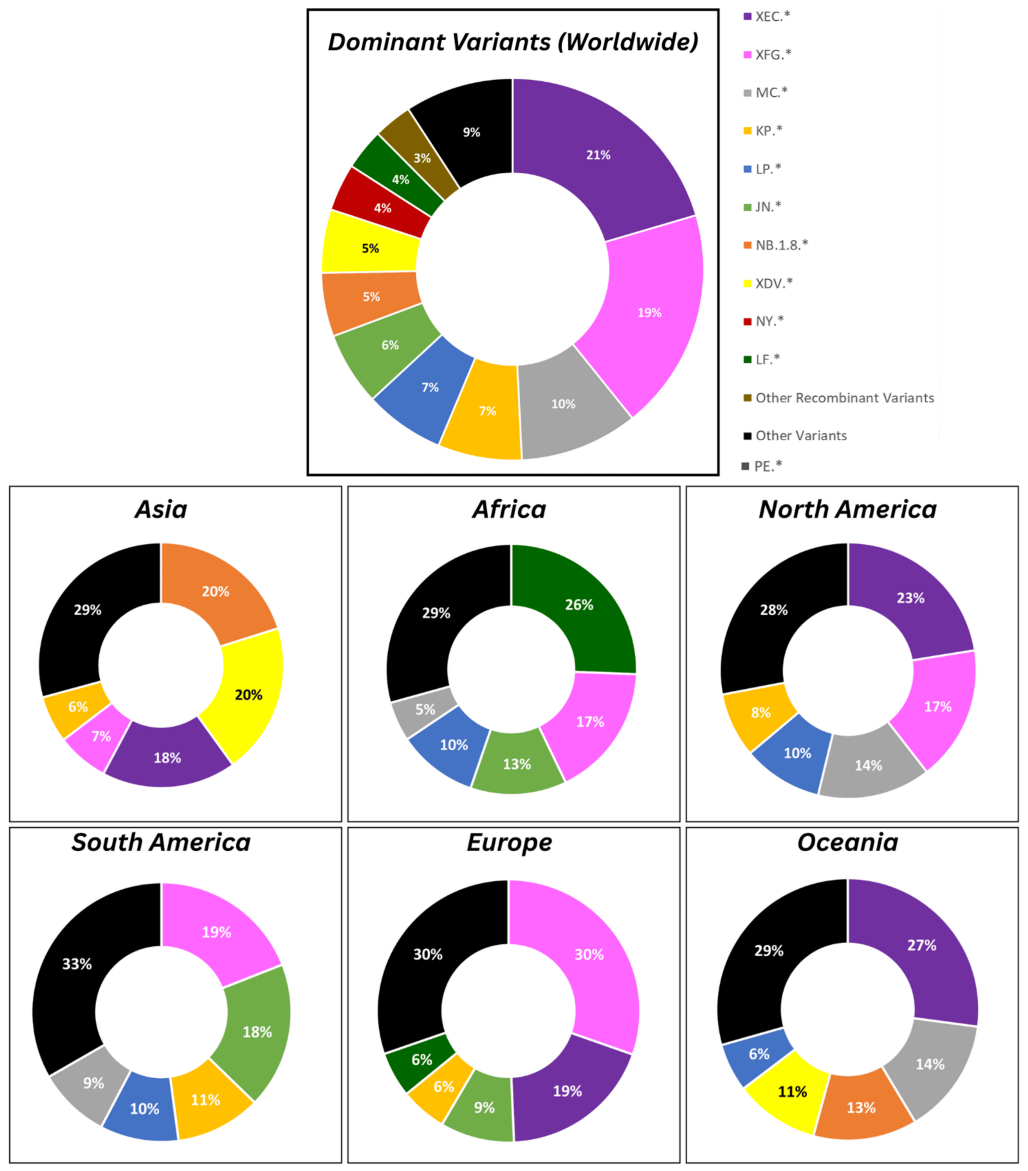
Global and continent-wise distribution of dominant SARS-CoV-2 variants

Regionally, variant predominance varied markedly by continent, highlighting localized circulation patterns (Figure 1). In Asia (n=27,385 sequences), NB.1.8 (20.1%) and XDV (19.9%) co-dominated with XEC (17.7%) and smaller shares from XFG (6.8%), and KP (6.2%). Africa contributed lower sequence volume (n=1,113 sequences) and greater fragmentation, led by LF (25.6%) and XFG (17.2%), followed by JN (12.4%), LP (10.3%), and other minor lineages. North America (n=98,071 sequences), the region with the highest submission rate, was driven by XEC (22.5%), XFG (17%), MC (14.3%), LP (10.1%), and smaller shares from KP.8 (8.2%), NY (5.5%), and JN (4.4%), reflecting diverse local dynamics. In South America (n=8,190 sequences), XFG (19%) prevailed, followed by JN (18.2%) and KP (10.6%). Europe (n=57,558 sequences) mirrored North American trends but with elevated XFG (30.4%) and XEC (19%), with shares from JN (9.1%), KP (5.7%), and LF (5.6%). Oceania (n=12,286 sequences) favored XEC (27.1%), MC (16.6%), NB.1.8 (12.8%), and XDV (10.4%), with XFG (5.1%) playing a smaller role.

These findings underscore the ongoing evolution and geographic heterogeneity of SARS-CoV-2, with XEC and XFG emerging as key global drivers in late 2025 globally, with continent-specific preferences, such as NB.1.8 in Asia and LF in Africa.

Temporal dynamics of SARS-CoV-2 variant circulation across continents

**Figure 2:**
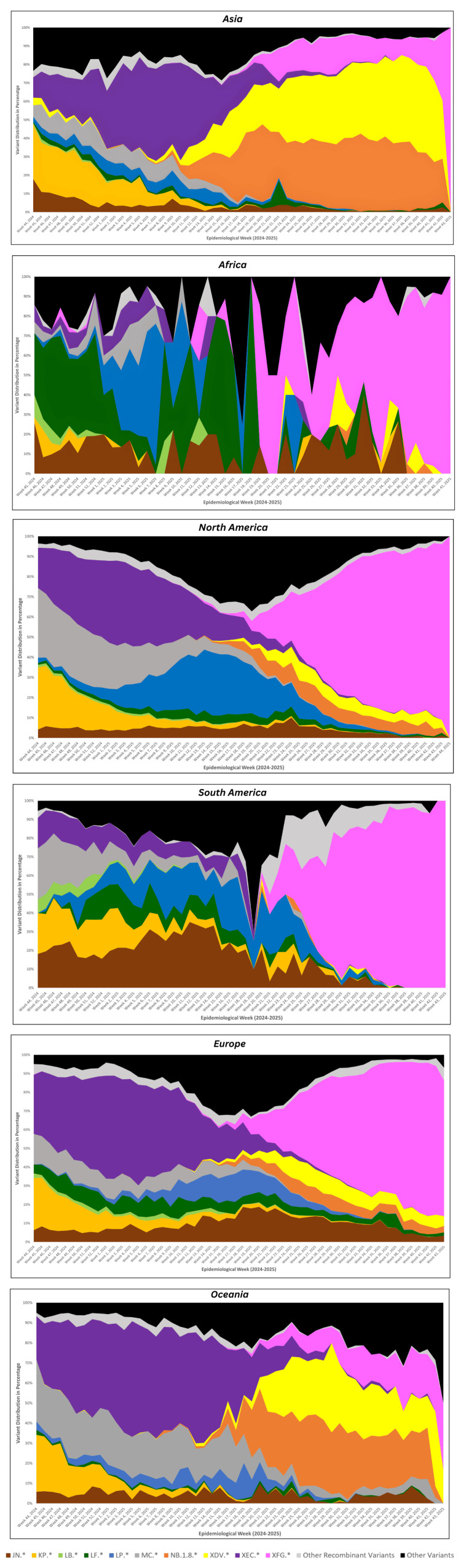
Temporal trends of major SARS-CoV-2 lineages circulating across continents

Figure 2 shows the spatiotemporal evolution of SARS-CoV-2 variants, weekly sequence data from 204,603 submissions (from week 44, 2024, to week 43, 2025). The data was stratified by continent and analyzed for proportional dominance across three periods: early (weeks 44-52, 2024), mid (weeks 1-26, 2025), and late (weeks 27-43, 2025). This revealed distinct regional trajectories amid global Omicron sublineage diversification, with declining submission volumes post-mid-2025 underscoring potential surveillance limitations.

Across continents, the circulating SARS-CoV-2 landscape showed substantial heterogeneity, yet several key patterns emerged. In Asia, early transmission was shaped primarily by KP and XEC, followed by a mid-phase shift towards XEC, NB.1.8, and XDV. XFG appeared later and increased steadily, reaching ~15% by the late phase, though it was not the dominant lineage in the region. In Africa, lineage patterns were more fragmented due to lower sampling. LF was the major contributor early on, transitioning to a more mixed profile during the mid-phase, with LP, LF, JN, and MC each contributing modestly. XFG remained sporadic until a late-phase spike (~69%), though this increase was driven by small sequence numbers and should be interpreted cautiously. In North America, lineage transitions were sharper. XEC and MC dominated early circulation, with LP gaining prominence mid-phase. XFG emerged slowly at first but expanded rapidly thereafter, ultimately accounting for more than 70% of late-phase genomes, representing one of the earliest and strongest regional takeovers by this lineage. South America exhibited a similar but even more pronounced pattern. Multiple variants co-circulated early (MC, JN, KP, and XEC), but XFG, initially absent, rose swiftly once introduced, becoming the overwhelmingly dominant lineage (~79%) in the late period and largely replacing other circulating variants. In Europe, early circulation was characterized by a strong presence of XEC. Although mid-phase diversity increased, XFG began to rise during this period and eventually became the predominant variant (~66%) in the late phase, marking a clear continental shift similar to that observed in the Americas. In Oceania, XEC and MC shaped early circulation, with increased lineage diversity mid-phase. XFG appeared later and expanded only modestly (~16%), never surpassing other dominant variants. This region, therefore, showed the slowest and least pronounced establishment of XFG globally. 

Across regions, XFG displayed a strikingly uneven pattern of global establishment. By the late phase, it had become the dominant lineage in North America, Europe, South America, and Africa, where it accounted for approximately 66-83% of all sequences, effectively replacing earlier circulating variants. In contrast, Asia and Oceania showed only modest increases (<20%). This coordinated rise across continents highlights the strong transmissibility and competitive fitness of XFG, consistent with rapid transcontinental spread.

Geographic distribution of the XFG variant 

Overall, XFG sequences constituted 18.63% of all SARS-CoV-2 genomes submitted globally to GISAID during the study period (38,114 XFG sequences out of 204,603 total genomes). The XFG variant was identified across all continents, demonstrating its broad geographical distribution. 

In absolute numbers, North America contributed the largest share of XFG genomes (43.3%; 16,513 out of 38,114 sequences), followed by Asia (4.9%; 1,869 out of 38,114 sequences) and South America (4.1%; 1,556 out of 38,114 sequences).

**Figure 3:**
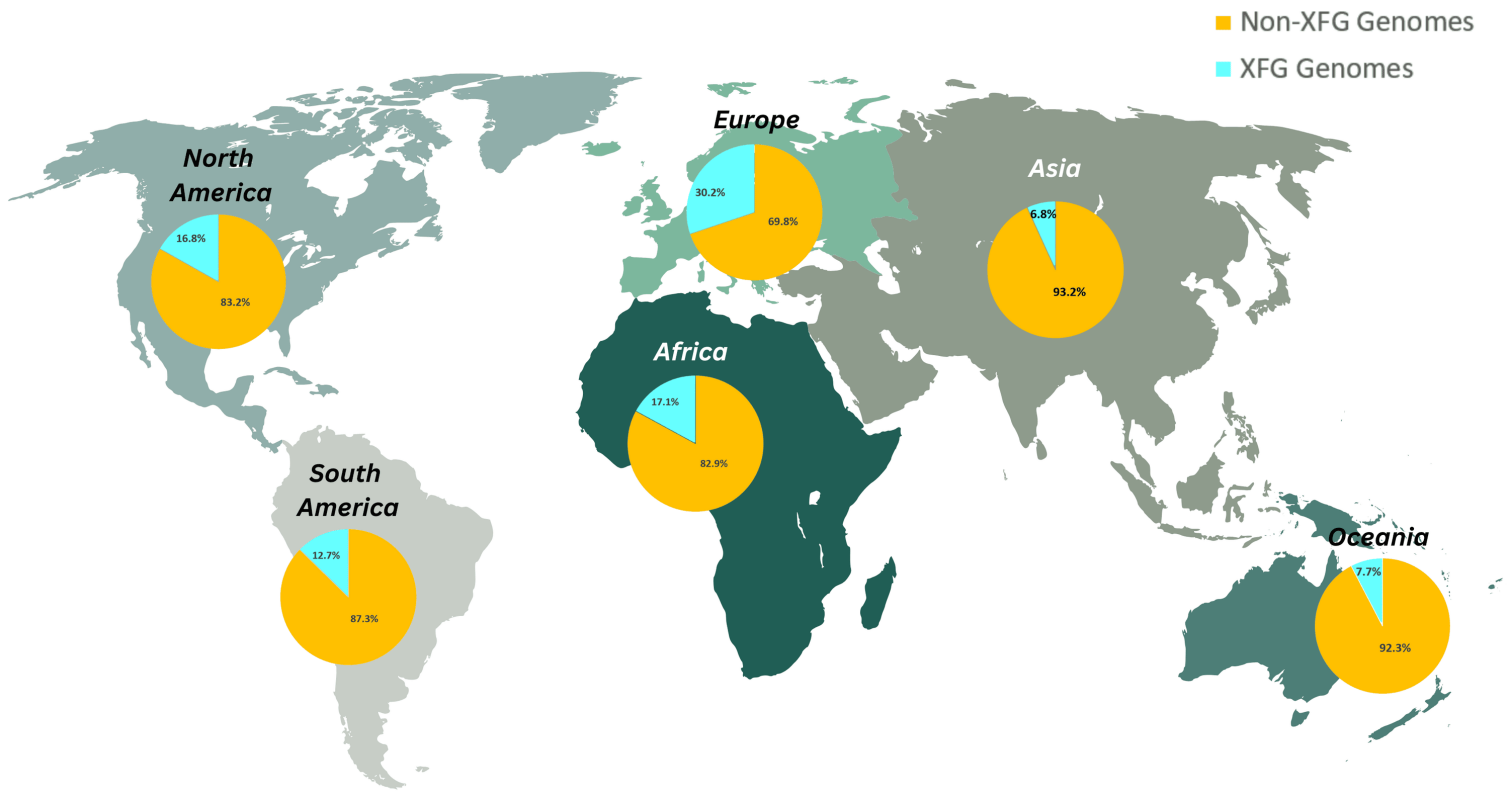
Global distribution of XFG variant sequences among submitted SARS-CoV-2 genomes

When assessed as a proportion of each continent's total XFG genome submissions, Europe showed the highest relative representation of XFG (30.2%), followed by Africa (17.1%), North America (16.8%), South America (12.7%), and Oceania (7.7%). In contrast, Asia recorded the lowest proportion, with XFG comprising only 6.8% of its total submitted genomes (Figure 3).

**Figure 4:**
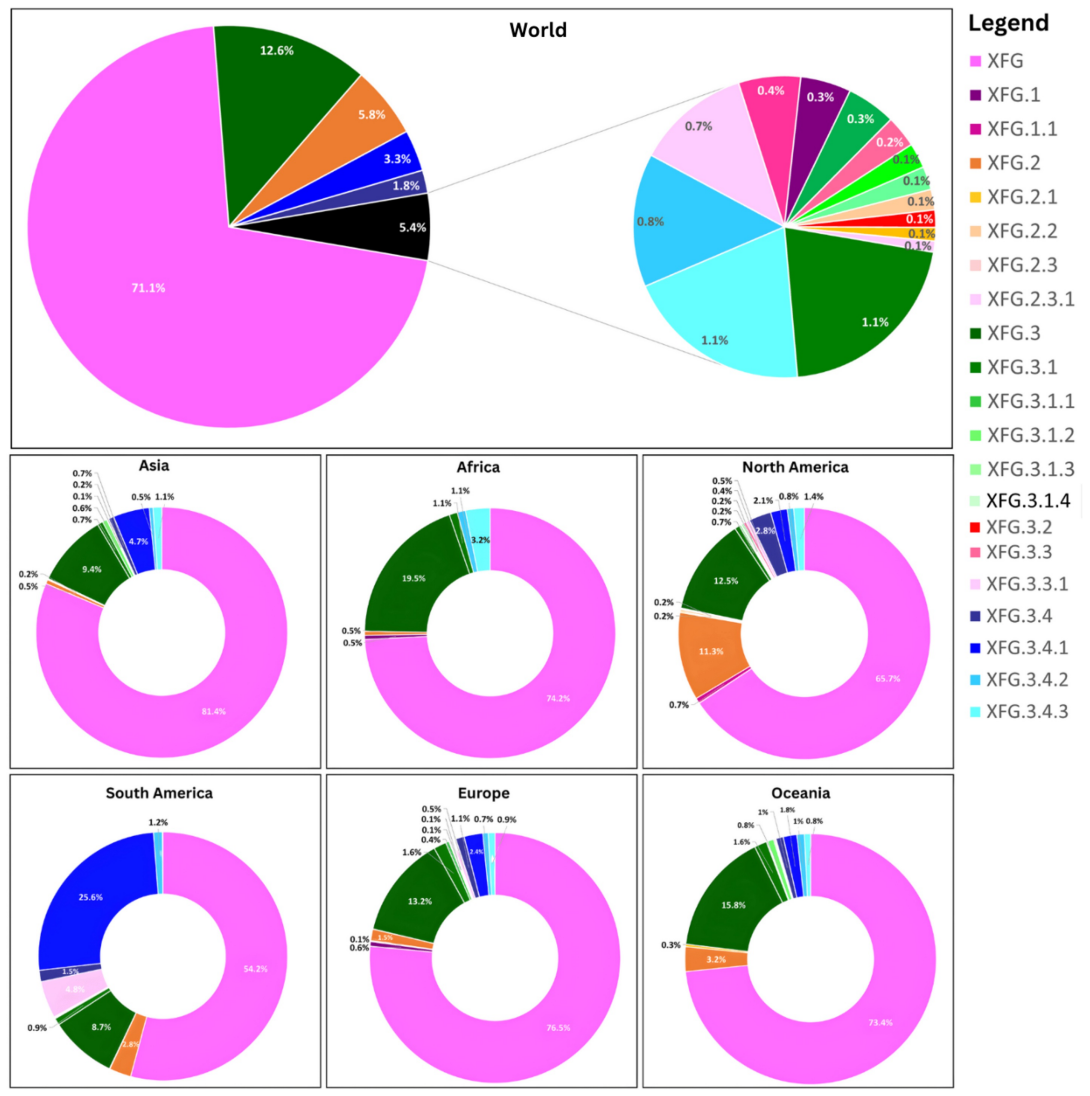
Global and continent-wise distribution of XFG and its sublineages

Analysis of 38,114 XFG genomes submitted globally revealed substantial sublineage diversification within the XFG lineage (Figure 4). At the global level, the parent lineage XFG accounted for 71.1% of all XFG genomes, followed by XFG.3 (12.6%), XFG.1 (5.8%), XFG.3.4.3 (5.4%), XFG.3.4 (3.3%), and XFG.2 (1.8%). Several minor sublineages, including XFG.1.1, XFG.2.1, XFG.2.2, XFG.3.1, XFG.3.1.1, and others, collectively constituted the remaining proportion (5.4%), each contributing ≤1% to the global dataset.

Across all continents, the parent lineage XFG was the dominant sublineage, comprising more than half of all XFG genomes in every region (ranging from 54.2% in South America to 81.4% in Asia). The second most prevalent sublineage globally was XFG.3, showing notable geographic variation, with the highest proportions observed in Africa (19.5%), Oceania (15.8%), and Europe (13.2%) and comparatively lower representation in Asia (9.4%), North America (12.5%), and South America (8.7%). Some regions exhibited additional locally enriched sublineages. South America showed the greatest diversification, with a substantial proportion of XFG.3.4.1 (25.6%) and smaller contributions from XFG.3.3.1 (4.8%). North America showed a higher representation of XFG.2 (11.3%), while Africa had notable proportions of XFG.3.4.3 (3.2%). In contrast, Europe and Oceania showed relatively limited sublineage diversity, with XFG and XFG.3 accounting for the majority of sequences.

Overall, these findings highlight both the global dominance of the XFG lineage and the heterogeneous expansion of its sublineages across continents, suggesting region-specific patterns of transmission and diversification.

Temporal trends in global XFG detection

**Figure 5:**
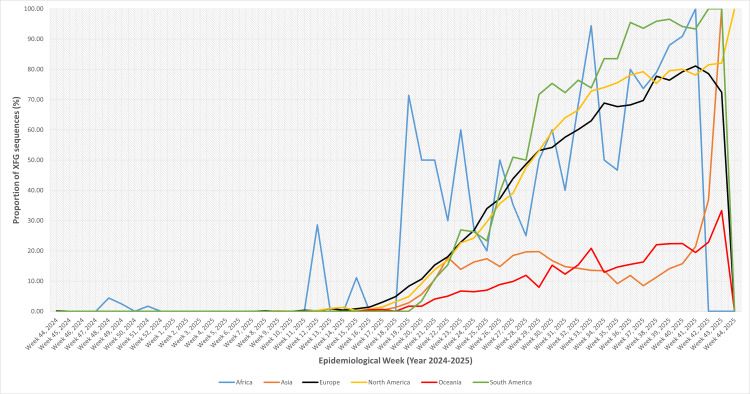
Weekly proportion of XFG sequences among total SARS-CoV-2 genomes submitted from each continent (week 44, 2024, to week 44, 2025)

Among the analyzed datasets, the earliest detection of the XFG lineage occurred in Europe, with one genome each from Spain (epidemiological week 44, 2024) and Russia (week 45, 2024) (Figure 5). Shortly thereafter, Africa (Kenya) detected its first XFG sequences in week 48, 2024, and North America (USA) recorded isolated detections in week 50, 2024. No XFG sequences were identified in Asia, Oceania, or South America during late 2024.

In early 2025, additional first detections appeared in Asia (Israel) (week 9, 2025). A consistent rise in XFG activity began around weeks 12-15, 2025, when several continents started reporting increasing numbers. This growth accelerated sharply from week 17 onward, with clear expansion across Europe, North America, and Asia and the first appearance in Oceania (a single genome from Australia in week 16, 2025). By week 20, a substantial increase was evident worldwide, particularly in Asia, Europe, North America, and Africa. South America, however, showed no activity until week 20 (a single genome from Brazil), marking a notably later emergence compared to other continents.

A period of sustained global increase extended from weeks 21 to 35, 2025, during which all continents exhibited steep increases in XFG submissions. Europe and North America experienced the most pronounced rises, peaking at week 36, 2025. In Asia, sequences peaked earlier at week 22, while Africa's activity remained lower but persistent, with weekly counts ranging from one to 22 cases. Oceania, despite its later emergence, demonstrated a steady rise from weeks 20 to 27, with peak activity in weeks 32-33, and South America, following its late introduction in week 20, showed a rapid increase with peaks between weeks 32 and 34.

Beginning week 37 onward, most continents exhibited a gradual decline, with sharp reductions observed after week 40. However, this downward trend must be interpreted cautiously, as the decrease coincides with a substantial reduction in the number of samples submitted for sequencing during this period, which may partially explain the observed fall in detected XFG sequences. By weeks 43-44, 2025, global XFG detection had markedly diminished, with only two cases reported worldwide in week 44.

In summary, XFG exhibited a globally synchronized but regionally staggered expansion, first detection in Europe and Africa in late 2024, followed by rapid amplification across all continents during mid-2025, including later-onset but substantial increases in Oceania and South America, and declining globally by late 2025.

Geographical and temporal distribution of the XFG SARS-CoV-2 variant in India

A total of 1,089 XFG sequences were retrieved from the IBDC portal and were included in the study. Following Nextclade Pangolin nomenclature, 27 different XFG sublineages were identified between week 44, 2024, and week 44, 2025. XFG.3 was the most common XFG sublineage (45.27%), followed by XFG.4 (41.78%) and XFG.5 (10.01%) (Table 1).

**Table 1:** Distribution of XFG and its sublineages in India based on sequences submitted to IBDC IBDC: Indian Biological Data Centre

XFG sublineages		Count		Percentage (%)
XFG		10		0.92
XFG.3	XFG.3	222	493	45.27
	XFG.3.1	4		
	XFG.3.1.2	27		
	XFG.3.10	2		
	XFG.3.16	59		
	XFG.3.18	1		
	XFG.3.3	2		
	XFG.3.4	44		
	XFG.3.4.1	8		
	XFG.3.4.2	12		
	XFG.3.4.3	92		
	XFG.3.5	3		
	XFG.3.5.1	8		
	XFG.3.7	9		
XFG.4	XFG.4	76	455	41.78
	XFG.4.1	342		
	XFG.4.2	37		
XFG.5	XFG.5	24	109	10.01
	XFG.5.1	84		
	XFG.5.2	1		
XFG.7		2		0.18
XFG.11		4		0.37
XFG.14		3		0.28
XFG.15		1		0.09
XFG.16		1		0.09
XFG.21		11		1.01
Grand total		1089		100

**Figure 6:**
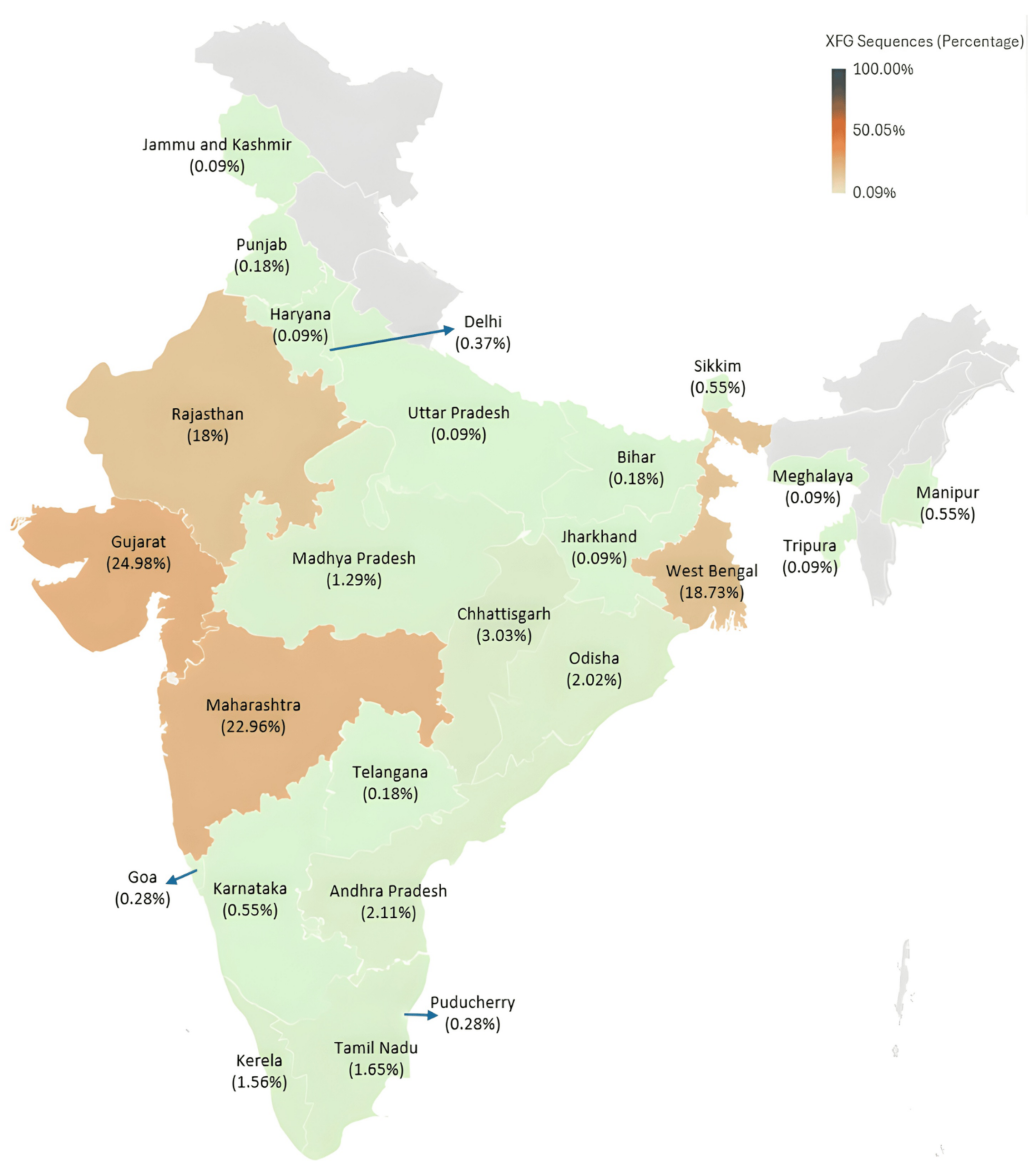
Geographic distribution of the XFG SARS-CoV-2 variant in India based on sequences submitted to IBDC IBDC: Indian Biological Data Centre

The XFG variant showed widespread geographic presence across India. The highest proportion of sequences was reported from Gujarat (272/1089, 24.98%), Maharashtra (250/1089, 22.96%), West Bengal (204/1089, 18.73%), and Rajasthan (196/1089, 18%), collectively accounting for over 80% of all XFG sequences detected nationwide (Figure 6).

**Figure 7:**
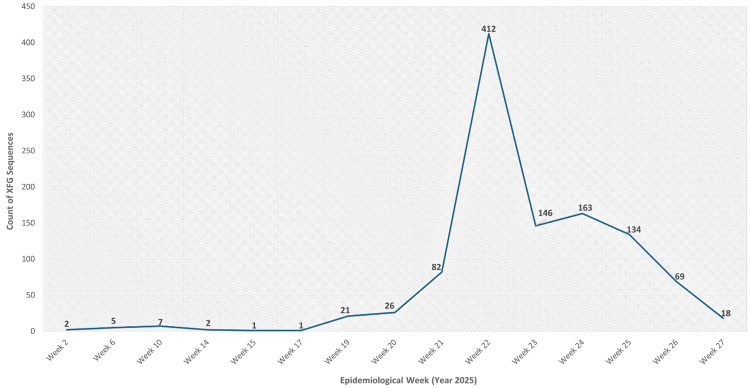
Temporal distribution of XFG variant sequences across India based on sequences submitted to IBDC IBDC: Indian Biological Data Centre

The first XFG sequence was detected in Maharashtra on January 6, 2025 (epidemiological week 2, 2025). Sporadic detections were observed until week 17 (late April 2025), followed by a gradual increase beginning in week 19 (early May 2025) and a sharp surge peaking at week 22 (late May 2025). Thereafter, the number of XFG sequences declined progressively through weeks 23-27 (June-July 2025) (Figure 7).

Epidemiological trends during the emergence of the XFG SARS-CoV-2 variant in Maharashtra, India

**Figure 8:**
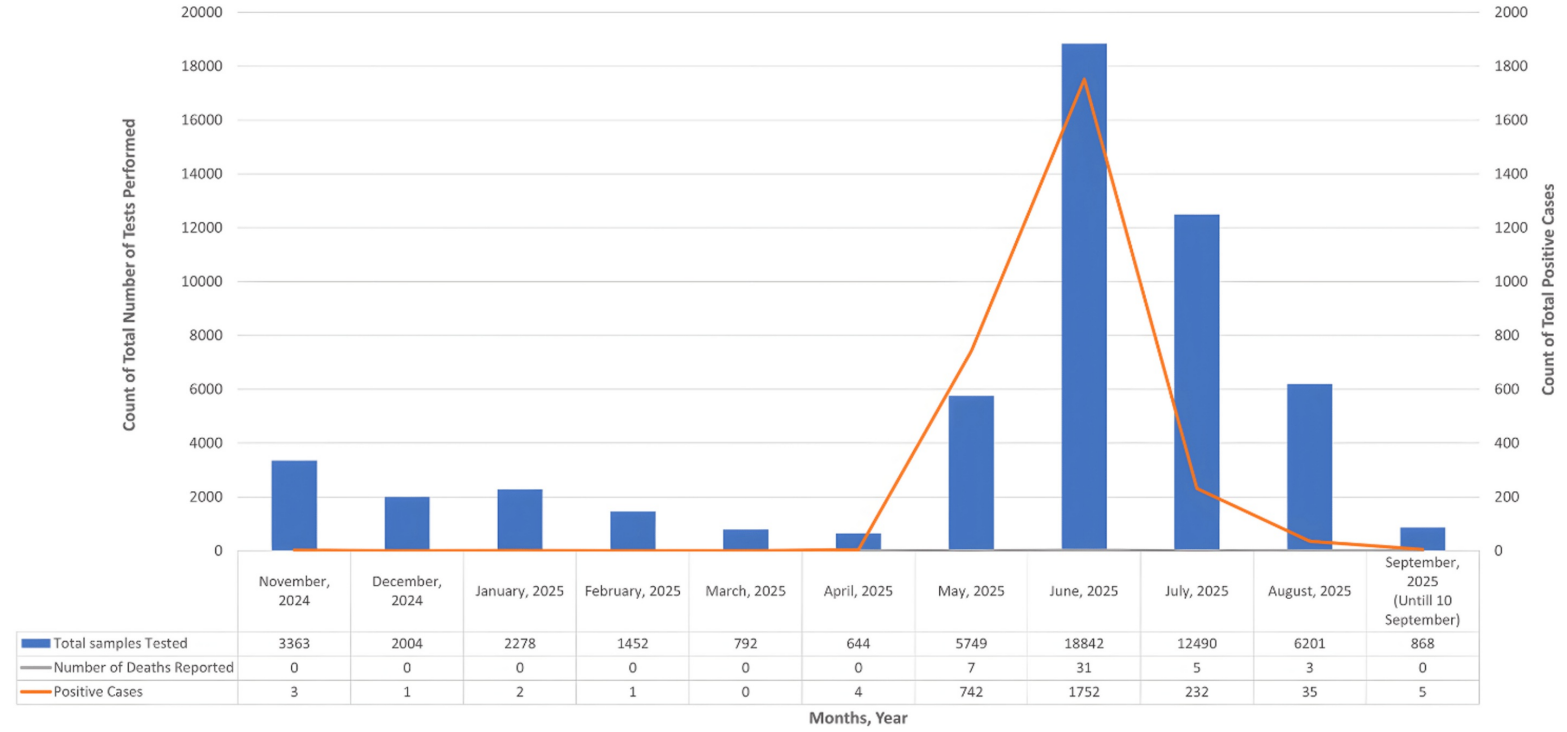
Trends in SARS-CoV-2 testing, positivity, and mortality in Maharashtra (November 2024 to September 10, 2025) Note: Deaths represent monthly reported counts and are included to indicate the low fatality observed during the XFG wave.

Between November 2024 and April 2025, SARS-CoV-2 activity remained minimal, with a case positivity rate below 1% and no reported deaths. A sharp rise in both case numbers and positivity was observed in May 2025 (12.9%) and June 2025 (9.3%) (Figure 8), coinciding with the emergence and rapid spread of the XFG variant. By July 2025, case positivity declined to 1.9% and continued to drop in subsequent months, aligning with the genomic evidence of a reduction in XFG detections. To assess the statistical significance of these changes, positivity rates were compared across aggregated periods using a chi-squared test: pre-May (November 2024-April 2025; rate=0.10%), peak period (May-July 2025; rate=7.35%), and post-July (August-September 2025; rate=0.57%). The results indicated highly significant differences in rates (χ²=1236.66; df=2; p<0.001), confirming that the observed increase during the peak period was statistically significant and not due to chance.

During this period, a parallel increase in discharge rates was noted, despite increased transmission, as evident in Figure 8. Although 46 deaths were reported between May and August 2025, the numbers remained low relative to the rise in case detections, suggesting that the XFG-associated increase in transmission did not translate into increased disease severity or fatality.

Overall, the epidemiological data from Maharashtra mirrors the genomic trend, demonstrating that the XFG variant exhibited a rapid and transient rise, driving a short-lived increase in case positivity without a proportional escalation in severe outcomes.

Demographic and clinical characteristics of the XFG SARS-CoV-2 variant in Maharashtra 

Complete metadata was available for 179 XFG cases included in the demographic study (Table 2). Among these 179 participants, 46.37% (83/179) were male, and 53.63% (96/179) were female. The median age of the study population was 38 (IQR: 28-60) years. The largest proportion of individuals belonged to the ≥60-year age group (24.58%). This was followed by individuals aged 30-39 years (21.79%) and 20-29 years (21.23%), together comprising over two-fifths of the study population. Most participants were from Pune (93.85%), followed by Ahilyanagar (1.68%) and Thane (1.12%).

**Table 2:** Demographic characteristics of the XFG SARS-CoV-2 variant in Maharashtra (n=179)

Demographic characteristics	Count (%)
Gender-wise distribution	
Male	83 (46.37%)
Female	96 (53.63%)
Age-wise distribution (years)	
0-9	9 (5.03%)
10-19	7 (3.91%)
20-29	38 (21.23%)
30-39	39 (21.79%)
40-49	23 (12.85%)
50-59	19 (10.61%)
60 and above	44 (24.58%)
Area-wise distribution in Maharashtra	
Pune	168 (93.85%)
Ahilyanagar	3 (1.68%)
Thane	2 (1.12%)
Sangli	1 (0.56%)
Satara	1 (0.56%)
Yavatmal	1 (0.56%)
Navi Mumbai	1 (0.56%)
Raigarh	1 (0.56%)
Latur	1 (0.56%)

All 179 identified cases were contacted for follow-up, of which 99 (55.31%) cases were included in the clinical study, while 80 (44.69%) cases could not be enrolled due to non-response, invalid contact details, or lack of consent. Table 3 summarizes the clinical characteristics, vaccination status, and outcome of XFG cases in Maharashtra. Most XFG cases had symptomatic disease (92 out of 99, 92.93%) with mild manifestations, such as fever, cough and cold, body ache, and fatigue. Underlying comorbid conditions, either alone or in combination, were reported in approximately one-third of cases (31 out of 99, 31.31%), with diabetes mellitus and hypertension emerging as the most prevalent, often co-occurring in affected individuals. Regarding the vaccination status, 90.91% (90 out of 99) of cases received at least one dose of the COVID-19 vaccine, while 9.09% (9 out of 99) were not vaccinated. All unvaccinated individuals were children aged 0-9 years. 

**Table 3:** Clinical characteristics of the XFG SARS-CoV-2 variant in Maharashtra (n=99)

Clinical characteristics			Count (%)
History of previous infection			
Yes			55 (55.56%)
No			44 (44.44%)
Presence of comorbidities			
Present (the comorbidities are grouped by system, and individual patients may have multiple comorbidities.)			31 (31.31%)
Metabolic disease	Diabetes mellitus		18 (58.06%)
	Dyslipidemia		2 (6.45%)
Cardiovascular disease	Hypertension		17 (54.84%)
	Hypotension		1 (3.23%)
	Underlying heart disease		2 (6.45%)
Endocrine disease	Hypothyroidism		2 (6.45%)
Renal disease	Renal failure		1 (3.23%)
Respiratory disease	Asthma		1 (3.23%)
Neurological disease	Parkinson's disease		1 (3.23%)
Autoimmune disease	Dermatomyositis		1 (3.23%)
Malignancy	Carcinoma		3 (9.68%)
Absent			68 (68.69%)
Vaccination status			
Vaccinated			90 (90.91%)
One dose			3 (3.33%)
Two doses			39 (43.33%)
Booster dose (precautionary dose)			48 (53.34%)
Not vaccinated			9 (9.09%)
Symptom status			
Symptomatic			92 (92.93%)
Asymptomatic			7 (7.07%)
Presenting symptoms (Presenting symptoms are grouped by system, and individual patients may have multiple symptoms.)			
Constitutional symptoms		Fever	75 (75.76%)
		Body ache	35 (35.35%)
		Fatigue	35 (35.35%)
Respiratory symptoms		Cold	39 (39.39%)
		Sore throat	29 (29.29%)
		Cough	47 (47.47%)
		Breathlessness	18 (18.18%)
Neurological symptoms		Headache	20 (20.20%)
		Loss of taste	12 (12.12%)
		Loss of smell	10 (10.10%)
Gastrointestinal symptoms		Vomiting	6 (6.06%)
		Diarrhea	5 (5.05%)
Type of isolation			
Home isolation			68 (68.69%)
Hospital isolation/hospitalization			31 (31.31%)
Type of treatment received			
Symptomatic treatment (antipyretics, antihistaminic, multivitamins)			82 (82.83%)
Need for oxygen therapy			13 (13.13%)
Oxygen therapy alone			8 (61.54%)
Invasive			2 (25%)
Non-invasive			6 (75%)
Oxygen therapy + steroid			1 (7.70%)
Oxygen therapy + antiviral treatment			2 (15.38%)
Oxygen therapy + antiviral treatment + steroid			2 (15.38%)
Antiviral treatment alone			2 (2.02%)
Steroids alone			2 (2.02%)
Outcome of disease			
Alive			95 (95.96%)
Dead			4 (4.04%)

Over two-thirds of patients (68 out of 99 cases, 68.69%) were managed at home with conservative symptomatic care, while the remainder (31 out of 99 cases, 31.31%) required hospitalization. Among the hospitalized cases, 61.29% (19 out of 31 cases) often received conservative treatment, with 38.71% of cases necessitating supplemental oxygen (12 out of 31 cases), often in non-invasive forms (8 out of 12 cases, 66.67%). The overall prognosis was favorable, with a near-complete recovery rate (95 out of 99 cases, 95.96%) and minimal mortality (4.04%), underscoring the generally benign course of disease in this cohort. All fatal cases occurred among elderly male patients (68-85 years) with significant underlying comorbidities. Two deaths were due to severe pneumonia and respiratory failure, while the remaining two deaths appeared to be related to decompensation of pre-existing conditions, including malignancy, renal failure, and acute cardiac events. In these cases, SARS-CoV-2 infection most likely appears to function as a precipitating factor, rather than the sole cause of mortality.

To identify factors associated with disease severity, bivariate analysis was performed using hospitalization (hospitalization vs. home isolation) and mortality as outcomes. The presence of comorbidities was significantly associated with both hospitalization (p=0.001) and mortality (p=0.008). In contrast, history of previous infection (p=0.66 for hospitalization; p=0.32 for mortality), vaccination status (p=0.46 for hospitalization; p=1 for mortality), and symptomatic presentation (p=1 for hospitalization; p=1 for mortality) were not significantly associated with either hospitalization or mortality.

On multivariate analysis, with hospitalization as outcome, the presence of comorbidities was the only independent predictor of hospitalization (adjusted odds ratio (aOR) 4.12; 95% CI 1.68-10.10; p=0.002). No associations were observed for vaccination status (p=0.63), previous infection (p=0.31), or symptomatic presentation (p=0.85). Owing to the small number of deaths (n=4) observed, multivariate analysis with mortality as an outcome was not performed.

## Discussion

The SARS-CoV-2 recombinant variant XFG, arising from recombination between LF.7 and LP.8.1.2, represents a notable evolutionary event during 2025. According to the WHO TAG-VE risk evaluation, XFG accounted for 22.7% of globally available sequences by epidemiological week 22 of 2025 (1,648 sequences from 38 countries), increasing sharply from 7.4% in week 19. This rise was observed across all WHO regions, with the most pronounced relative increase seen in the Southeast Asia region, where XFG rapidly replaced the previously dominant NB.1.8.1. However, in India, XFG emerged as the predominant circulating variant during spring 2025, while NB.1.8.1 remained uncommon [5], highlighting marked regional heterogeneity in variant replacement dynamics. Consistent with this expansion, as of January 2026, XFG demonstrates an estimated global relative growth advantage of approximately 19% [7].

The XFG variant carries a characteristic set of spike mutations compared to NB.1.8.1, including S31P, K182R, R190S, R346T, K444R, V445R, T478K, N487D, and T572I. When compared to the JN.1 variant, XFG displays the following spike mutations: T22N, S31P, K182R, R190S, R346T, K444R, V445R, F456L, N487D, Q493E, and T572I [5,1]. However, these mutations alone cannot establish heightened risk and must be interpreted in the context of prevalence and genomic background. Branda et al. used phylogenomic and phylodynamic analyses to show that the Bayesian Skyline Plot and Lineages-Through-Time trajectories of the XFG variant are consistent with a brief but intense expansion (during late March-April), followed by evolutionary stabilization (from May to July), indicative of a currently non-threatening variant [1]. This pattern suggests that the lineage took an extended time to acquire its key mutations, a behavior that is typically not observed in variants of concern. Furthermore, the estimated evolutionary rate for XFG (~4.4×10⁻⁴ substitutions per site per year) is comparable to other recent SARS-CoV-2 variants and is substantially lower than the early pandemic strains, supporting the interpretation of endemic-era evolution rather than a lineage with unusually accelerated evolutionary potential [1].

Further, multiple recent reports suggest that the rapid expansion of XFG is plausibly driven primarily by enhanced immune escape, rather than markedly improved receptor binding. In a comprehensive pseudovirus neutralization and ACE2 inhibition study of dominant JN.1 subvariants, XFG and NB.1.8.1 demonstrated a clear growth advantage over previously dominant LP.8.1.1 and showed lower serum neutralization titers compared with LP.8.1.1 across cohorts, with XFG exhibiting an approximately 1.9-fold reduction in geometric mean neutralizing titers in KP.2 monovalent vaccine recipients and significantly reduced titers in non-boosted individuals, supporting increased antibody evasion as a key correlate of dominance [8]. These findings are concordant with an independent post-KP.2 vaccination immunogenicity analysis, where neutralizing titers against XFG were markedly reduced (GMT ~17) relative to vaccine-matched KP.2 and ancestral WA.1 and antigenic cartography positioned emerging FLiRT-associated variants, including XFG, at substantial antigenic distances from KP.2, consistent with immune escape potential that may erode population-level neutralization [9]. XFG also showed ~2-fold lower NT50 in convalescent plasma from inactivated vaccine recipients with JN.1/XDV infections, compared to LP.8.1.1 [10]. Structurally, the spike of XFG harbors additional RBD changes (including H445R, N487D, and Q493E, along with T572I in SD1). The monoclonal antibody profiling suggests that XFG resistance is attributable to N487D and Q493E, in escape from class 1/2 (group B) antibodies [8,10]. Notably, despite this immune-evasive phenotype, all three studies indicate a trade-off with receptor engagement, reporting reduced ACE2-binding/engagement efficiency for XFG relative to LP.8.1.1 [8-10]. It is likely linked to structural effects of mutations such as N487D, which may introduce electrostatic repulsion at the ACE2 interface. These mutations likely promote an "RBD down" conformation, enhancing evasion despite 2.34-fold lower ACE2-binding affinity (higher IC50) and reduced pseudovirus entry vs. LP.8.1.1 [8,10]. Compensatory changes like R346T and T572I in SD1 may stabilize the spike, countering fitness costs and enabling XFG's transmission advantage [8]. Taken together, these data support an evolutionary trajectory in which XFG appears to have gained a transmission advantage chiefly through immune escape, potentially enabling widespread dissemination, an interpretation that is consistent with our observation of rapid spread.

In the present study, the clinical disease associated with the XFG variant was predominantly mild, with limited progression to severe outcomes. Our findings closely align with findings from a recent community-based surveillance study in the Netherlands, comparing SARS-CoV-2 variants circulating during 2024 (dominated by the KP.3 variant) and 2025 (dominated by the XFG variant). The study showed that XFG infection did not confer increased clinical severity compared to earlier Omicron-descendant variants such as KP.3. The symptom burden and overall disease severity, assessed using adjusted health scores and total symptom counts, were comparable between the two periods, reinforcing the absence of intrinsically more severe disease associated with XFG [11]. Among the hospitalized patients in our cohort, most required only conservative management, with supplemental oxygen, predominantly non-invasive, needed in a minority. Mortality was low and confined to elderly individuals with significant underlying comorbidities, suggesting that SARS-CoV-2 infection likely acted as a precipitating or aggravating factor rather than the sole cause of death, consistent with the evolving understanding of COVID-19 outcomes in vulnerable populations. The predominance of mild illness and favorable clinical outcomes in our cohort support the conclusion that XFG infection is not associated with increased disease severity. It is noteworthy that bivariate and multivariate analyses of hospitalized patients showed a significant association with comorbidities. 

This study has several limitations. First, the analysis was dependent on the SARS-CoV-2 sequences uploaded to public databases, which are influenced by regional sequencing capacity, submission practices, and reporting delays, potentially introducing sampling bias. In addition, the findings are based on individuals who underwent SARS-CoV-2 testing, had their samples sequenced, and consented to participate, which may under-represent asymptomatic or untested infections and limit estimation of the true disease burden. As the clinical analysis was restricted to cases from Maharashtra, the generalizability of the findings to other regions with differing demographic and epidemiological profiles may be limited. Also, clinical data were collected through telephonic interviews and may be subject to recall/reporting bias. Furthermore, non-response may also influence estimates of disease severity. Thus, future studies incorporating broader geographic coverage, standardized sequencing efforts, and prospective clinical data collection would improve the representativeness and accuracy of assessments of emerging SARS-CoV-2 variants.

## Conclusions

The XFG variant has emerged as the dominant SARS-CoV-2 variant globally during late 2024 and 2025, driving transient waves across the continents. In India, XFG circulated widely across multiple states, with a short-lived surge during mid-2025. In Maharashtra, where genomic trends closely paralleled epidemiological patterns, the infections were predominantly mild, manifesting as fever, cough, cold, body ache, and fatigue. Despite its attenuated virulence, the variant's rapid transcontinental spread and sublineage diversification underscore SARS-CoV-2's enduring immune evasion and adaptability. Fluctuations in testing volumes and sequencing submissions may underestimate the true burden of infection. This emphasizes the need for continued surveillance for the early detection of emerging variants and for strengthening preparedness against future waves.

## Figures and Tables

**Figure 9:**
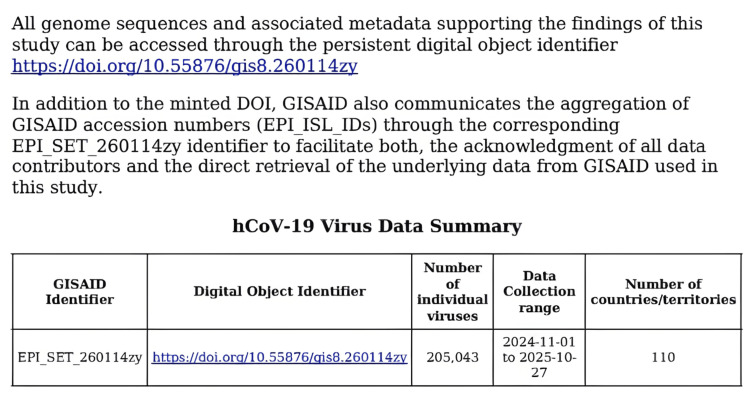
Details of sequences retrieved from the Global Initiative on Sharing All Influenza Data (GISAID)

## Appendices

Appendix A

Appendix B

**Table 4:** IDs of the sequences retrieved from the Indian Biological Data Centre (IBDC) Retrieved from the Indian Biological Data Centre (IBDC), Regional Centre for Biotechnology, Department of Biotechnology, Ministry of Science and Technology, Government of India, with their permission

IBDC ID
INCOV296930
INCOV296931
INCOV296939
INCOV296940
INCOV296959
INCOV296962
INCOV296964
INCOV296966
INCOV296967
INCOV296969
INCOV296972
INCOV296974
INCOV296984
INCOV296988
INCOV296992
INCOV296998
INCOV297000
INCOV297002
INCOV297003
INCOV297005
INCOV297007
INCOV297011
INCOV297013
INCOV297017
INCOV297018
INCOV297024
INCOV297026
INCOV297031
INCOV297032
INCOV297035
INCOV297037
INCOV297038
INCOV297039
INCOV297042
INCOV297043
INCOV297044
INCOV297045
INCOV297046
INCOV297048
INCOV297049
INCOV297050
INCOV297051
INCOV297053
INCOV297054
INCOV297055
INCOV297056
INCOV297057
INCOV297058
INCOV297060
INCOV297062
INCOV297063
INCOV297064
INCOV297066
INCOV297067
INCOV297069
INCOV297072
INCOV297073
INCOV297077
INCOV297085
INCOV297086
INCOV297087
INCOV297089
INCOV297092
INCOV297097
INCOV297107
INCOV297108
INCOV297109
INCOV297111
INCOV297112
INCOV297114
INCOV297115
INCOV297117
INCOV297118
INCOV297120
INCOV297126
INCOV297127
INCOV297130
INCOV297135
INCOV297137
INCOV297138
INCOV297139
INCOV297144
INCOV297145
INCOV297146
INCOV297147
INCOV297148
INCOV297149
INCOV297150
INCOV297151
INCOV297152
INCOV297153
INCOV297154
INCOV297155
INCOV297156
INCOV297159
INCOV297161
INCOV297163
INCOV297164
INCOV297165
INCOV297166
INCOV297168
INCOV297169
INCOV297173
INCOV297174
INCOV297176
INCOV297178
INCOV297179
INCOV297180
INCOV297185
INCOV297192
INCOV297193
INCOV297194
INCOV297195
INCOV297197
INCOV297200
INCOV297201
INCOV297204
INCOV297205
INCOV297206
INCOV297207
INCOV297210
INCOV297211
INCOV297212
INCOV297213
INCOV297214
INCOV297215
INCOV297217
INCOV297218
INCOV297219
INCOV297221
INCOV297222
INCOV297223
INCOV297225
INCOV297226
INCOV297229
INCOV297230
INCOV297231
INCOV297234
INCOV297236
INCOV297238
INCOV297239
INCOV297240
INCOV297241
INCOV297242
INCOV297243
INCOV297245
INCOV297247
INCOV297249
INCOV297250
INCOV297251
INCOV297256
INCOV297257
INCOV297258
INCOV297259
INCOV297260
INCOV297261
INCOV297262
INCOV297263
INCOV297264
INCOV297266
INCOV297267
INCOV297268
INCOV297269
INCOV297270
INCOV297272
INCOV297273
INCOV297274
INCOV297275
INCOV297278
INCOV297279
INCOV297280
INCOV297285
INCOV297287
INCOV297289
INCOV297293
INCOV297294
INCOV297295
INCOV297296
INCOV297297
INCOV297301
INCOV297302
INCOV297303
INCOV297305
INCOV297306
INCOV297308
INCOV297312
INCOV297313
INCOV297314
INCOV297315
INCOV297316
INCOV297321
INCOV297322
INCOV297323
INCOV297324
INCOV297325
INCOV297333
INCOV297335
INCOV297340
INCOV297341
INCOV297342
INCOV297345
INCOV297346
INCOV297347
INCOV297350
INCOV297351
INCOV297355
INCOV297356
INCOV297361
INCOV297362
INCOV297366
INCOV297367
INCOV297369
INCOV297370
INCOV297371
INCOV297372
INCOV297374
INCOV297376
INCOV297377
INCOV297378
INCOV297379
INCOV297386
INCOV297389
INCOV297390
INCOV297393
INCOV297395
INCOV297396
INCOV297398
INCOV297400
INCOV297401
INCOV297404
INCOV297405
INCOV297408
INCOV297409
INCOV297410
INCOV297411
INCOV297415
INCOV297416
INCOV297417
INCOV297418
INCOV297421
INCOV297423
INCOV297431
INCOV297435
INCOV297438
INCOV297809
INCOV297810
INCOV297812
INCOV297813
INCOV297814
INCOV297815
INCOV297816
INCOV297817
INCOV297819
INCOV297820
INCOV297822
INCOV297823
INCOV297825
INCOV297827
INCOV297829
INCOV297831
INCOV297832
INCOV297833
INCOV297834
INCOV297837
INCOV297838
INCOV297839
INCOV297840
INCOV297843
INCOV297845
INCOV297848
INCOV297849
INCOV297852
INCOV297853
INCOV297854
INCOV297856
INCOV297857
INCOV297858
INCOV297859
INCOV297860
INCOV297861
INCOV297862
INCOV297864
INCOV297865
INCOV297866
INCOV297867
INCOV297868
INCOV297869
INCOV297874
INCOV297875
INCOV297876
INCOV297877
INCOV297878
INCOV297884
INCOV297885
INCOV297886
INCOV297887
INCOV297890
INCOV297891
INCOV297893
INCOV297894
INCOV297896
INCOV297897
INCOV297899
INCOV297900
INCOV297902
INCOV297903
INCOV297904
INCOV297905
INCOV297906
INCOV297908
INCOV297910
INCOV297911
INCOV297912
INCOV297913
INCOV297914
INCOV297916
INCOV297917
INCOV297920
INCOV297921
INCOV297923
INCOV297926
INCOV297928
INCOV297929
INCOV297930
INCOV297931
INCOV297932
INCOV297934
INCOV297935
INCOV297936
INCOV297938
INCOV297939
INCOV297941
INCOV297942
INCOV297943
INCOV297944
INCOV297945
INCOV297947
INCOV297948
INCOV297950
INCOV297951
INCOV297952
INCOV297953
INCOV297954
INCOV297955
INCOV297956
INCOV297957
INCOV297959
INCOV297960
INCOV297962
INCOV297963
INCOV297964
INCOV297965
INCOV297966
INCOV297967
INCOV297968
INCOV297970
INCOV297971
INCOV297972
INCOV297974
INCOV297975
INCOV297976
INCOV297977
INCOV297978
INCOV297979
INCOV297980
INCOV297981
INCOV297982
INCOV297983
INCOV297984
INCOV297987
INCOV297988
INCOV297989
INCOV297990
INCOV297991
INCOV297992
INCOV297993
INCOV297994
INCOV297995
INCOV297996
INCOV297998
INCOV298000
INCOV298002
INCOV298003
INCOV298004
INCOV298005
INCOV298007
INCOV298008
INCOV298009
INCOV298010
INCOV298011
INCOV298012
INCOV298013
INCOV298014
INCOV298017
INCOV298018
INCOV298019
INCOV298020
INCOV298021
INCOV298022
INCOV298023
INCOV298024
INCOV298025
INCOV298026
INCOV298027
INCOV298029
INCOV298030
INCOV298033
INCOV298034
INCOV298035
INCOV298036
INCOV298037
INCOV298039
INCOV298040
INCOV298041
INCOV298042
INCOV298044
INCOV298045
INCOV298047
INCOV298048
INCOV298050
INCOV298052
INCOV298053
INCOV298055
INCOV298056
INCOV298057
INCOV298059
INCOV298060
INCOV298061
INCOV298063
INCOV298064
INCOV298067
INCOV298068
INCOV298069
INCOV298070
INCOV298073
INCOV298074
INCOV298077
INCOV298078
INCOV298079
INCOV298080
INCOV298081
INCOV298082
INCOV298083
INCOV298085
INCOV298087
INCOV298088
INCOV298089
INCOV298091
INCOV298093
INCOV298094
INCOV298095
INCOV298096
INCOV298098
INCOV298100
INCOV298102
INCOV298103
INCOV298105
INCOV298106
INCOV298107
INCOV298108
INCOV298110
INCOV298111
INCOV298112
INCOV298113
INCOV298114
INCOV298115
INCOV298116
INCOV298118
INCOV298119
INCOV298121
INCOV298122
INCOV298123
INCOV298125
INCOV298126
INCOV298127
INCOV298129
INCOV298130
INCOV298131
INCOV298132
INCOV298133
INCOV298134
INCOV298135
INCOV298136
INCOV298138
INCOV298139
INCOV298143
INCOV298144
INCOV298146
INCOV298147
INCOV298148
INCOV298153
INCOV298155
INCOV298156
INCOV298159
INCOV298160
INCOV298161
INCOV298162
INCOV298165
INCOV298166
INCOV298167
INCOV298168
INCOV298170
INCOV298171
INCOV298172
INCOV298173
INCOV298174
INCOV298176
INCOV298177
INCOV298178
INCOV298222
INCOV298224
INCOV298225
INCOV298227
INCOV298228
INCOV298229
INCOV298230
INCOV298231
INCOV298232
INCOV298233
INCOV298236
INCOV298237
INCOV298238
INCOV298239
INCOV298240
INCOV298241
INCOV298242
INCOV298243
INCOV298244
INCOV298245
INCOV298246
INCOV298247
INCOV298248
INCOV298249
INCOV298250
INCOV298251
INCOV298252
INCOV298255
INCOV298256
INCOV298257
INCOV298262
INCOV298263
INCOV298265
INCOV298269
INCOV298270
INCOV298272
INCOV298273
INCOV298274
INCOV298275
INCOV298276
INCOV298278
INCOV298279
INCOV298283
INCOV298285
INCOV298291
INCOV298296
INCOV298297
INCOV298302
INCOV298304
INCOV298305
INCOV298308
INCOV298314
INCOV298315
INCOV298321
INCOV298324
INCOV298325
INCOV298336
INCOV298338
INCOV298342
INCOV298344
INCOV298345
INCOV298349
INCOV298350
INCOV298351
INCOV298352
INCOV298358
INCOV298363
INCOV298364
INCOV298366
INCOV298369
INCOV298370
INCOV298371
INCOV298372
INCOV298373
INCOV298374
INCOV298376
INCOV298377
INCOV298379
INCOV298380
INCOV298381
INCOV298382
INCOV298385
INCOV298386
INCOV298389
INCOV298390
INCOV298393
INCOV298395
INCOV298397
INCOV298398
INCOV298399
INCOV298401
INCOV298402
INCOV298403
INCOV298404
INCOV298405
INCOV298406
INCOV298407
INCOV298408
INCOV298409
INCOV298410
INCOV298412
INCOV298415
INCOV298416
INCOV298417
INCOV298418
INCOV298420
INCOV298422
INCOV298428
INCOV298429
INCOV298430
INCOV298431
INCOV298433
INCOV298435
INCOV298436
INCOV298437
INCOV298438
INCOV298440
INCOV298442
INCOV298443
INCOV298444
INCOV298446
INCOV298449
INCOV298451
INCOV298452
INCOV298454
INCOV298455
INCOV298456
INCOV298457
INCOV298459
INCOV298462
INCOV298463
INCOV298466
INCOV298468
INCOV298470
INCOV298473
INCOV298474
INCOV298476
INCOV298478
INCOV298479
INCOV298481
INCOV298482
INCOV298483
INCOV298485
INCOV298488
INCOV298489
INCOV298490
INCOV298491
INCOV298492
INCOV298495
INCOV298497
INCOV298498
INCOV298499
INCOV298500
INCOV298505
INCOV298506
INCOV298510
INCOV298517
INCOV298519
INCOV298523
INCOV298524
INCOV298526
INCOV298527
INCOV298528
INCOV298529
INCOV298531
INCOV298533
INCOV298535
INCOV298536
INCOV298537
INCOV298539
INCOV298540
INCOV298541
INCOV298544
INCOV298545
INCOV298548
INCOV298550
INCOV298551
INCOV298554
INCOV298555
INCOV298556
INCOV298563
INCOV298564
INCOV298565
INCOV298567
INCOV298568
INCOV298571
INCOV298576
INCOV298577
INCOV298578
INCOV298580
INCOV298582
INCOV298584
INCOV298585
INCOV298588
INCOV298589
INCOV298590
INCOV298591
INCOV298592
INCOV298593
INCOV298594
INCOV298595
INCOV298597
INCOV298598
INCOV298600
INCOV298603
INCOV298604
INCOV298605
INCOV298607
INCOV298609
INCOV298612
INCOV298613
INCOV298614
INCOV298619
INCOV298620
INCOV298621
INCOV298625
INCOV298626
INCOV298627
INCOV298629
INCOV298630
INCOV298631
INCOV298663
INCOV298664
INCOV298684
INCOV298686
INCOV298689
INCOV298690
INCOV298691
INCOV298692
INCOV298693
INCOV298695
INCOV298696
INCOV298698
INCOV298699
INCOV298702
INCOV298704
INCOV298705
INCOV298708
INCOV298712
INCOV298719
INCOV298720
INCOV298721
INCOV298722
INCOV298729
INCOV298732
INCOV298733
INCOV298742
INCOV298743
INCOV298744
INCOV298745
INCOV298746
INCOV298747
INCOV298748
INCOV298750
INCOV298752
INCOV298754
INCOV298755
INCOV298756
INCOV298757
INCOV298758
INCOV298759
INCOV298764
INCOV298767
INCOV298768
INCOV298769
INCOV298770
INCOV298771
INCOV298772
INCOV298773
INCOV298774
INCOV298775
INCOV298776
INCOV298777
INCOV298778
INCOV298779
INCOV298780
INCOV298781
INCOV298782
INCOV298783
INCOV298784
INCOV298785
INCOV298786
INCOV298788
INCOV298789
INCOV298790
INCOV298791
INCOV298792
INCOV298796
INCOV298801
INCOV298803
INCOV298805
INCOV298806
INCOV298807
INCOV298809
INCOV298810
INCOV298812
INCOV298813
INCOV298814
INCOV298815
INCOV298816
INCOV298817
INCOV298818
INCOV298819
INCOV298820
INCOV298822
INCOV298824
INCOV298825
INCOV298826
INCOV298828
INCOV298829
INCOV298830
INCOV298831
INCOV298832
INCOV298833
INCOV298835
INCOV298836
INCOV298837
INCOV298840
INCOV298841
INCOV298842
INCOV298843
INCOV298844
INCOV298845
INCOV298846
INCOV298848
INCOV298849
INCOV298850
INCOV298851
INCOV298852
INCOV298853
INCOV298858
INCOV298859
INCOV298861
INCOV298867
INCOV298869
INCOV298870
INCOV298873
INCOV298874
INCOV298875
INCOV298877
INCOV298879
INCOV298880
INCOV298883
INCOV298884
INCOV298885
INCOV298888
INCOV298889
INCOV298890
INCOV298891
INCOV298892
INCOV298893
INCOV298894
INCOV298899
INCOV298900
INCOV298901
INCOV298902
INCOV298903
INCOV298904
INCOV298905
INCOV298906
INCOV298908
INCOV298910
INCOV298911
INCOV298912
INCOV298913
INCOV298914
INCOV298916
INCOV298919
INCOV298921
INCOV298924
INCOV298927
INCOV298929
INCOV298930
INCOV298931
INCOV298932
INCOV298934
INCOV298935
INCOV298936
INCOV298938
INCOV298939
INCOV298940
INCOV298942
INCOV298943
INCOV298944
INCOV298947
INCOV298949
INCOV298950
INCOV298951
INCOV298953
INCOV298954
INCOV298955
INCOV298956
INCOV298957
INCOV298958
INCOV298959
INCOV298960
INCOV298961
INCOV298962
INCOV298963
INCOV298967
INCOV298969
INCOV298971
INCOV298972
INCOV298974
INCOV298975
INCOV298977
INCOV298978
INCOV298980
INCOV298982
INCOV298984
INCOV298985
INCOV298986
INCOV298987
INCOV298988
INCOV298989
INCOV298990
INCOV298992
INCOV298995
INCOV299000
INCOV299002
INCOV299005
INCOV299007
INCOV299009
INCOV299010
INCOV299013
INCOV299014
INCOV299016
INCOV299018
INCOV299020
INCOV299023
INCOV299024
INCOV299026
INCOV299027
INCOV299028
INCOV299030
INCOV299034
INCOV299035
INCOV299037
INCOV299038
INCOV299039
INCOV299042
INCOV299044
INCOV299045
INCOV299046
INCOV299047
INCOV299049
INCOV299050
INCOV299052
INCOV299053
INCOV299054
INCOV299056
INCOV299059
INCOV299061
INCOV299062
INCOV299064
INCOV299065
INCOV299068
INCOV299069
INCOV299072
INCOV299073
INCOV299075
INCOV299079
INCOV299081
INCOV299089
INCOV299091
INCOV299092
INCOV299094
INCOV299095
INCOV299096
INCOV299097
INCOV299100
INCOV299101
INCOV299103
INCOV299105
INCOV299106
INCOV299107
INCOV299108
INCOV299109
INCOV299110
INCOV299112
INCOV299113
INCOV299114
INCOV299115
INCOV299117
INCOV299118
INCOV299119
INCOV299121
INCOV299122
INCOV299123
INCOV299124
INCOV299125
INCOV299127
INCOV299128
INCOV299131
INCOV299132
INCOV299135
INCOV299137
INCOV299138
INCOV299139
INCOV299140
INCOV299145
INCOV299146
INCOV299147
INCOV299148
INCOV299149
INCOV299150
INCOV299151
INCOV299153
INCOV299154
INCOV299155
INCOV299157
INCOV299159
INCOV299161
INCOV299164
INCOV299165
INCOV299166
INCOV299167
INCOV299179
INCOV299184
INCOV299187
INCOV299188
INCOV299189
INCOV299190
INCOV299191
INCOV299192
INCOV299193
INCOV299194
INCOV299195
INCOV299196
INCOV299197
INCOV299198
INCOV299199
INCOV299200
INCOV299201
INCOV299202
INCOV299203
INCOV299204
INCOV299205
INCOV299207
INCOV299209
INCOV299211
INCOV299212
INCOV299214
INCOV299216
INCOV299218
INCOV299223
INCOV299226
INCOV299227
INCOV299229
INCOV299230
INCOV299231
INCOV299233
INCOV299234
INCOV299235
INCOV299239
INCOV299240
INCOV299241
INCOV299242
INCOV299244
INCOV299245
INCOV299246
INCOV299248
INCOV299249
INCOV299251
INCOV299252
INCOV299254
INCOV299255
INCOV299256
INCOV299258
INCOV299259
INCOV299260
INCOV299261
INCOV299262
INCOV299265
INCOV299267
INCOV299268
INCOV299269
INCOV299271
INCOV299273
INCOV299274

Appendix C

**Table 5:** Questionnaire template used for the telephonic interview

Questionnaire	
Demographic data	
1. Sample ID	
2. Patient name	
3. Gender	
4. Age (in years)	
5. Phone number	
6. District of residence	
7. Sample collection date	
8. SARS-CoV-2 variant	
Clinical data	
1. Presence of symptoms	1. Present 2. Absent
a. Fever	1. Yes 2. No
b. Cold	1. Yes 2. No
c. Cough	1. Yes 2. No
d. Sore throat	1. Yes 2. No
e. Breathlessness	1. Yes 2. No
f. Headache	1. Yes 2. No
g. Runny nose	1. Yes 2. No
h. Weakness	1. Yes 2. No
i. Myalgia	1. Yes 2. No
j. Body ache	1. Yes 2. No
k. Vomiting	1. Yes 2. No
l. Diarrhea	1. Yes 2. No
m. Loss of taste	1. Yes 2. No
n. Loss of smell	1. Yes 2. No
o. Other symptoms (if any)	
2. Presence of comorbidities	1. Present 2. Absent
What comorbidities present	
3. Home isolation	1. Yes 2. No
4. Hospitalization/institutional quarantine	1. Yes 2. No
5. Oxygen requirement	1. Yes 2. No
a. Non-invasive	1. Yes 2. No
b. Invasive (intubation)	1. Yes 2. No
6. History of previous infection	1. Yes 2. No
7. Travel history	1. Yes 2. No
8. Vaccine taken	1. Yes 2. No 3. Not applicable
9. Vaccine name	1. Covishield 2. Covaxin 3. Pfizer 4. Others
a. Dose 1 taken	1. Yes 2. No
b. Dose 2 taken	1. Yes 2. No
c. Booster (precautionary) dose taken	1. Yes 2. No
10. Type of treatment received?	1. Symptomatic (antipyretics, multivitamins, antihistaminic) 2. Oxygen therapy 3. Steroids 4. Antivirals 5. Monoclonal antibodies 6. Others (specify)
11. Outcome	1. Alive 2. Dead
